# Associations between informal care costs, care quality, carer rewards, burden and subsequent grief: the international, access, rights and empowerment mortality follow-back study of the last 3 months of life (IARE I study)

**DOI:** 10.1186/s12916-020-01768-7

**Published:** 2020-11-03

**Authors:** Irene J. Higginson, Deokhee Yi, Bridget M. Johnston, Karen Ryan, Regina McQuillan, Lucy Selman, Stephen Z. Pantilat, Barbara A. Daveson, R. Sean Morrison, Charles Normand

**Affiliations:** 1 Department of Palliative Care, Cicely Saunders Institute of Palliative Care, Policy & Rehabilitation, King’s College London, Bessemer Road, London, SE5 9PJ UK; 2 King’s College Hospital Foundation Trust, Bessemer Road, London, SE5 9PJ UK; 3 The Centre of Health Policy and Management, Trinity College Dublin, Room 0.21, 3-4 Foster Place, College Green, Dublin 2, Ireland; 4 Mater Misericordiae Hospital, Eccles Street, Dublin 7, Ireland; 5 Beaumont Hospital, Beaumont Road, Dublin 9, Ireland; 6 Population Health Sciences, Bristol Medical School, University of Bristol, Bristol, UK; 7 Palliative Care Program, Division of Hospital Medicine, Department of Medicine, University of California, San Francisco, San Francisco, CA USA; 8 Brookdale Department of Geriatrics and Palliative Medicine, Icahn School of Medicine at Mount Sinai, New York, NY USA

**Keywords:** Informal care, End of life, Costs of care, Mortality follow-back survey, Grief, Carer burden

## Abstract

**Background:**

At the end of life, formal care costs are high. Informal care (IC) costs, and their effects on outcomes, are not known. This study aimed to determine the IC costs for older adults in the last 3 months of life, and their relationships with outcomes, adjusting for care quality.

**Methods:**

Mortality follow-back postal survey.

Setting: Palliative care services in England (London), Ireland (Dublin) and the USA (New York, San Francisco).

Participants: Informal carers (ICrs) of decedents who had received palliative care.

Data: ICrs reported hours and activities, care quality, positive aspects and burdens of caregiving, and completed the Texas Revised Inventory of Grief (TRIG).

Analysis: All costs (formal, informal) were calculated by multiplying reported hours of activities by country-specific costs for that activity. IC costs used country-specific shadow prices, e.g. average hourly wages and unit costs for nursing care. Multivariable logistic regression analysis explored the association of potential explanatory variables, including IC costs and care quality, on three outcomes: positive aspects and burdens of caregiving, and subsequent grief.

**Results:**

We received 767 completed surveys, 245 from London, 282 Dublin, 131 New York and 109 San Francisco. Most respondents were women (70%); average age was 60 years. On average, patients received 66–76 h per week from ICrs for ‘being on call’, 52–55 h for ICrs being with them, 19–21 h for personal care, 17–21 h for household tasks, 15–18 h for medical procedures and 7–10 h for appointments. Mean (SD) IC costs were as follows: USA $32,468 (28,578), England $36,170 (31,104) and Ireland $43,760 (36,930). IC costs accounted for 58% of total (formal plus informal) costs. Higher IC costs were associated with less grief and more positive perspectives of caregiving. Poor home care was associated with greater caregiver burden.

**Conclusions:**

Costs to informal carers are larger than those to formal care services for people in the last three months of life. If well supported ICrs can play a role in providing care, and this can be done without detriment to them, providing that they are helped. Improving community palliative care and informal carer support should be a focus for future investment.

## Background

In palliative care, those important to the patient, such as family members and informal carers (hereafter called ‘informal carers’, ICrs) are part of the unit of care. ICrs often provide high levels of demanding care and support willingly, because they see this as part of their relationship with the patient and are ambivalent to considering their own needs [1]. While some ICrs report positive outcomes such as closer relationships with others, greater appreciation of life, increased empathy and positive self-view, many can experience anxiety, depression, decline in quality of life and/or post-traumatic stress disorder [2]. Patient symptoms, such as breathlessness, fatigue or cognitive impairments, and advanced illness can increase caregiver burden and/or reduce caregiver rewards [3–6]. However, this research is usually based on small, single setting studies [3–6].

Formal care costs in the last year of life account for between 10 and 25% of health care costs [7–10], but to the best of our knowledge, informal care costs have never been compared internationally. Their costs are much less recognised or understood [11]. Systematic review evidence indicates that living with relatives and/or extended family support doubles or more (up to 7.8 times) the odds of patients being able to be cared for and to die at home, something for which many people wish [12]. The activities of ICrs likely save states billions in health and social care spending each year [13]. Home palliative care is thought to be cost-effective [14, 15]. But what are the costs for ICrs? Dying at home when preferred can often require informal caregivers to be given time off work [16], which is unpaid. The relationship of IC costs with the outcomes for carers, such as burden, or subsequent grief are not known.

Therefore, as part of the International, Access, Rights, and Empowerment (IARE I) study of palliative care in three countries, we aimed to determine and compare the informal care (IC) costs and their associations with self-reported caregiver burden, rewards and subsequent caregiver grief, taking account of care quality, as reported by ICrs.

## Methods

### Study design

We conducted a mortality follow-back postal survey of key informants (normally relatives and informal carers) of decedents identified by palliative care services in participating hospitals. Reporting follows STROBE [17] and MORECARE statements [18]. See declarations for ethical approvals. Further details are provided elsewhere [19].

### Settings

Three countries included are in the top 10 (of 80 countries) of the Economist Intelligence Unit Quality of Death Index; rankings (scores) are as follows: England − 1 (93.9), Ireland − 4 (85.8) and the USA − 9 (80.8) [20]. This index ranks the quality of palliative and end of life care across the world according to predetermined criteria, national data and interviews. The countries had different health care systems (England: National Health Service; Ireland: National Health Insurance; USA: Private Health System, palliative care covered by most insurance agencies and Medicare and Medicaid [21]) and philanthropy supporting hospice and palliative care [22]. Participating palliative care services in London (England), Dublin (Ireland), New York and San Francisco (USA) were as follows: established hospital palliative care consulting teams in all countries, a hospital-based community outreach team in London and an inpatient palliative care ward in New York. Details of the participating services are found elsewhere [19, 23, 24].

### Inclusion criteria

We identified patients aged ≥ 65 years who had accessed (≥ 1 contact) a participating palliative care team and died 4–10 months prior to the survey date. Their next of kin, as indicated in clinical records, was sent study information and a postal questionnaire from their clinical service (following data-protection regulations), with a pre-paid envelope addressed to the research team. The next of kin was asked to complete the questionnaire or pass it to the most appropriate individual who was close to the patient for completion. All data were analysed anonymously.

### Questionnaire and data collection

Consenting respondents returned a self-completed questionnaire, pre-piloted in all countries. Respondents reported demographic data including socio-economic status, living arrangements and relationship to patient and the patient’s illnesses. This was supplemented by patient record data on age, diagnosis and co-morbidities. In addition, ICrs reported health and social care services used by patients in the last 3 months of life.

Informal time spent caring was counted with the Client Service Receipt Inventory (CSRI) [19, 23], by asking respondents to document all IC time spent by family and friends as well as the respondents during the last 3 months of the patient’s life. Six questions covered a wide range of possible physical, social, emotional and other caring activities, including time spent ‘on call’ and being available for the patient (Additional file 1: Table S1). Answers were given as categories of hours per week: less than 5 h, 5–9 h, 10–19 h, 20–49 h, 50 or more hours and all the time.

Quality of care of the last place of stay (e.g. hospital, home) in the last 3 months was rated using Likert scales from 1 (very poor) to 6 (excellent).

Carer burden and positive aspects of caregiving (PAC) at the time when patients died was measured according to the ZARIT 12 [25, 26] and a set of eight questions derived from previous studies, respectively [4, 27, 28].

Subsequent grief was assessed using the revised Texas Revised Inventory of Grief (TRIG). This measures the intensity of grief after the death of a close person and has two scales: TRIG I (past behaviours when patient died, eight items) and TRIG II (present feelings, referred to as subsequent grief in this study, 13 items) [29–31].

### Analysis

Hours of IC spent per week for each item were converted as the middle point of the given range: 2.5 for less than 5 h, 7 for 5–9 h and 15, 30, 50 and 112 for the rest. To determine the IC costs for each patient, we multiplied the number of hours of care with country-specific shadow prices such as average hourly wages and unit costs for nursing care. All costs were translated into USD ($) for comparison, using the purchasing power parity (PPP) index. We checked the summary statistics and plotted the informal cost distributions of all patients in each country for illustrative purposes.

Formal care costs were extracted from an earlier analysis on this dataset and are presented to aid interpretation. These were calculated by multiplying the quantity of specific services used according to the CSRI with corresponding country-specific unit costs [19].

#### Descriptive analyses

We described provision and hours of IC by country. We explored the distribution of formal health and social care costs (in $1000) and IC costs (in $1000) and calculated the proportion of IC costs in the total societal costs. We also described the IC costs by carers’ relationship to the patients. After examining the distribution of subsequent grief, carer burden and PAC by country and carer’s relationship to the patient, we plotted the univariate relationship between these variables and IC costs.

#### Regression analyses

We examined the factors associated with subsequent grief, carer burden and PAC using multiple regression analysis. We selected explanatory variables based on previous literature reviews, meta-analysis and theoretical considerations [2, 32, 33]. These included age, gender, patient’s cause of death (cancer or not), carer’s relationship to patient, a religious faith of carer, carer’s feeling about household financial status, carer’s quality rating with care at hospital or home and informal and formal care costs. Country fixed effects were also included in the models. We used complete cases only.

### Sample size

We calculated the sample size based on being able to detect a difference in the mean IC costs between countries, with 80% of power and *α* = 0.05 (0.025 with Bonferroni correction for two pairs of comparison), which would require 229 individuals in each country.

## Results

### Sample characteristics

We received 767 completed surveys: 245 (32.4%) of 756 delivered surveys in London, 282/580 (48.6%) in Dublin, 131/548 (23.9%) in New York and 109/342 (31.9%) in San Francisco. Missing values were infrequent (2–5% of variables), if any, and scattered with no patterns.

#### Carers

Most respondents were women (70%) and average age was 60 years (Table 1). 34.7% of all respondents were daughter of patients, followed by wife or female partner (22.4%), husband or male partner (12.3%) and son (11.7%). In Ireland, 92.6% of respondents had a religious belief, which is higher than in the UK (82.6%) and the USA (79.9%). About 2/3 were living comfortably or doing alright regarding the household income of themselves. More carers were in paid employment in the USA (52.4%) than in the UK (35.3%) or Ireland (43.6%).

**Table 1:** Characteristics of the deceased patients and respondents (bereaved carer) (unit: %, mean (s.d.))

	London (*N* = 245)	Ireland (*N* = 282)	USA (*N* = 240)	All (*N* = 767)
**Bereaved carer**				
Women (*n* = 749)	72.6%	67.9%	70.9%	70.4%
Age (*n* = 738)	61.6(12.6)	59.2(13.7)	60.9(14.1)	60.5(13.5)
Ethnicity (= 1 if white) (*n* = 725)	85.2%	100.0%	59.3%	82.5%
Employment status (*n* = 730)				
Employed and paid	35.3%	43.6%	52.4%	43.7%
Employed and unpaid	8.5%	13.6%	8.7%	10.4%
Unemployed	8.9%	9.1%	6.5%	8.2%
Retired	47.2%	33.7%	32.5%	37.7%
Relationship (*n* = 742)				
Wife or female partner	22.1%	20.5%	27.4%	22.4%
Husband or male partner	12.1%	11.9%	14.1%	12.3%
Daughter	37.5%	38.4%	31.2%	34.7%
Son	9.6%	16.0%	10.3%	11.7%
Female other	8.8%	7.1%	8.6%	7.8%
Male other	2.5%	4.5%	2.6%	3.1%
Others	7.5%	1.5%	6.0%	4.7%
Household income (*n* = 750)				
Living comfortably	31.0%	19.2%	45.4%	31.2%
Doing alright	37.6%	44.3%	32.5%	38.5%
Just about getting by	9.8%	16.3%	8.8%	11.9%
Finding it quite difficult	9.4	10.6%	3.8%	8.1%
Finding it very difficult	2.5%	0.7%	0.8%	1.3%
Do not know	2.5%	2.5%	4.2%	3.0%
Religious belief (= 1 if yes) (*N* = 729)	82.6%	92.6%	79.9%	85.5%
ZARIT				
Burden^1)^ (*N* = 734)	25.1(8.8)	23.6(9.4)	24.6(8.7)	24.4(9.0)
Positive aspects of caregiving^2)^ (*N* = 729)	29.2(6.9)	30.0(7.4)	30.7(6.7)	30.0(7.1)
TRIG				
Past behaviour (*N* = 734)	20.5(8.9)	21.7(8.3)	20.1(7.4)	20.8(8.3)
Subsequent feeling (*N* = 738) ^3)^	43.0(13.8)	44.7(13.1)	41.1(12.0)	43.0(13.1)
**Patient**				
Women	54.3%	51.4%	52.9%	52.8%
Age (years) (*n* = 766)	79.7(8.3)	80.8(8.2)	78.5(9.1)	79.7(8.6)
65–69	13.9%	12.8%	21.7%	15.9%
70–79	36.3%	29.9%	32.9%	32.9%
80–89	36.3%	41.6%	33.3%	37.3%
90–102	13.5%	15.7%	12.1%	13.8%
Diagnosis (*n* = 763)				
Lung and respiratory cancer	13.5%	11.4%	7.9%	11.0%
Breast cancer	2.0%	3.9%	2.9%	3.0%
Genitourinary cancer	12.7%	7.5%	5.8%	8.6%
Lymphatic cancer	10.2%	5.7%	5.8%	7.2%
Digestive cancer	12.2%	10.3%	12.1%	11.5%
Ill-defined cancer	6.1%	3.9%	0%	3.4%
Other cancer	2.9%	5.0%	4.2%	4.0%
Non-cancer respiratory	7.4%	16.0%	9.2%	11.1%
Non-cancer circulatory	14.7%	16.7%	26.3%	19.0%
Non-cancer CNS^4)^	10.2%	7.1%	2.9%	6.8%
Renal failure	1.2%	3.2%	6.7%	3.7%
Other non-cancer^4)^	5.7%	9.2%	16.3%	10.3%
Primary carer (= 1 if available) (*n* = 731)	87.6%	87.1%	92.2%	88.8%
Living with (= 1 if yes) (*n* = 746)	61.4%	67.9%	75.2%	68.1%
Religious belief (= 1 if religious)	88.1%	98.5%	84.6%	90.8%
Number of carers (*n* = 731)	2.5(1.8)	3.0(2.0)	2.6(1.7)	2.7(1.9)
Health and social care costs (USD)	15,756(15,036)	29,210(24,231)	37,327(37,234)	27,452(28,203)

Twelve items had scales from 0 to 4 (0 never; 1 rarely; 2 sometimes; 3 quite frequently; 4 nearly always), and the range of total scores is 0 to 48. Higher score means more distressful burden

Eight items had scales from 0 to 4 (0 never; 1 rarely; 2 sometimes; 3 quite frequently; 4 nearly always), and the range of total scores is 0 to 32. Higher score means more positive feeling about caregiving experience

Revised Texas Revised Inventory of Grief. Past behaviour and subsequent grief sums 8 and 13 items respectively where each item was measured in Likert scale (1 completely true to 5 completely false). Higher scores are indicative of less psychological distress

Non-cancer CNS included Alzheimer’s dementia, Parkinson’s related disorders, motor neurone disease, multiple sclerosis and other neurological diseases. Note that some people with dementia may have been coded as ‘other non-cancer’

Subsequent grief was the summation of 13 items of TRIG, with mean scores of 20.8 (SD 8.3) and 43.0 (SD 13.1). Mean scores of ZARIT 12 measuring carer’s burden were 24.4 (SD 9.0). Positive aspects of caregiving (PAC) were measured using 8 items and its mean score was 30.0 (SD 7.1).

#### Patients

The average age of those who had died was 80 years, with similar numbers of women and men (Table 1). Patients had on average 2–3 ICrs; 68% had an ICr living with them; 49% had cancer as a cause of death. Health and social care costs were on average $27,452 (SD $28,203), highest in the USA ($37,327) and lowest in the UK ($15,756).

### Hours of informal care (IC)

The most common care giving activities in all countries were spending time with the patient (82–89% of patients received this) and ‘being on call’ (78–81% of patients received this), i.e. being there to watch for problems at least a few hours per week (Table 2). Household tasks were provided to 67–69% of patients. More than half of the patients were helped with personal care and medical procedures. On average, patients received 19–21 h of IC per week from friends or family for personal care, 15–18 h for medical procedure, 7–10 h for appointments and 17–21 h for household tasks. Friends and/or family spent 66–76 h per week on being on call and 52–55 h with patients.

**Table 2:** Number (percentage) of patients in the last 3 months of life who received informal care (IC) support according to main categories in the CSRI questionnaire, and the mean (SD) hours provided per week provided by ICrs for each of these activities

	England (*N* = 245)				Ireland (*N* = 282)				USA (*N* = 240)			
	*n*		Mean	s.d.	*n*		Mean	s.d.	*n*		Mean	s.d.
Personal care	141	57.6%	19.12	18.62	163	57.8%	21.02	18.64	131	54.6%	18.59	17.65
Medical procedure	135	55.1%	17.02	18.03	154	54.6%	18.49	18.77	135	56.3%	15.05	16.61
Appointments	170	69.4%	7.57	10.54	190	67.4%	7.30	10.47	164	68.3%	9.82	11.75
Household task	182	74.3%	21.24	17.55	190	67.4%	20.88	17.63	168	70.0%	16.72	15.52
On call	199	81.2%	76.00	46.12	221	78.4%	72.83	46.63	187	77.9%	66.23	48.48
Time with patient	201	82.0%	54.88	47.12	251	89.0%	53.02	46.38	206	85.8%	51.94	45.75

Notes: The questions used in the questionnaires were as follows: Did you and other friends or family help with (1) personal care? (e.g. washing, dressing), (2) medical procedures? (e.g. taking medicines), (3) going to appointments or treatments?, (4) household tasks? (e.g. shopping, cooking), (5) time spent on call (i.e. being with her/him if needed) and (6) time spent with her/him (e.g. visiting, doing things together)

### Informal care (IC) costs versus formal care costs and by relationship to patient

**Fig. 1:**
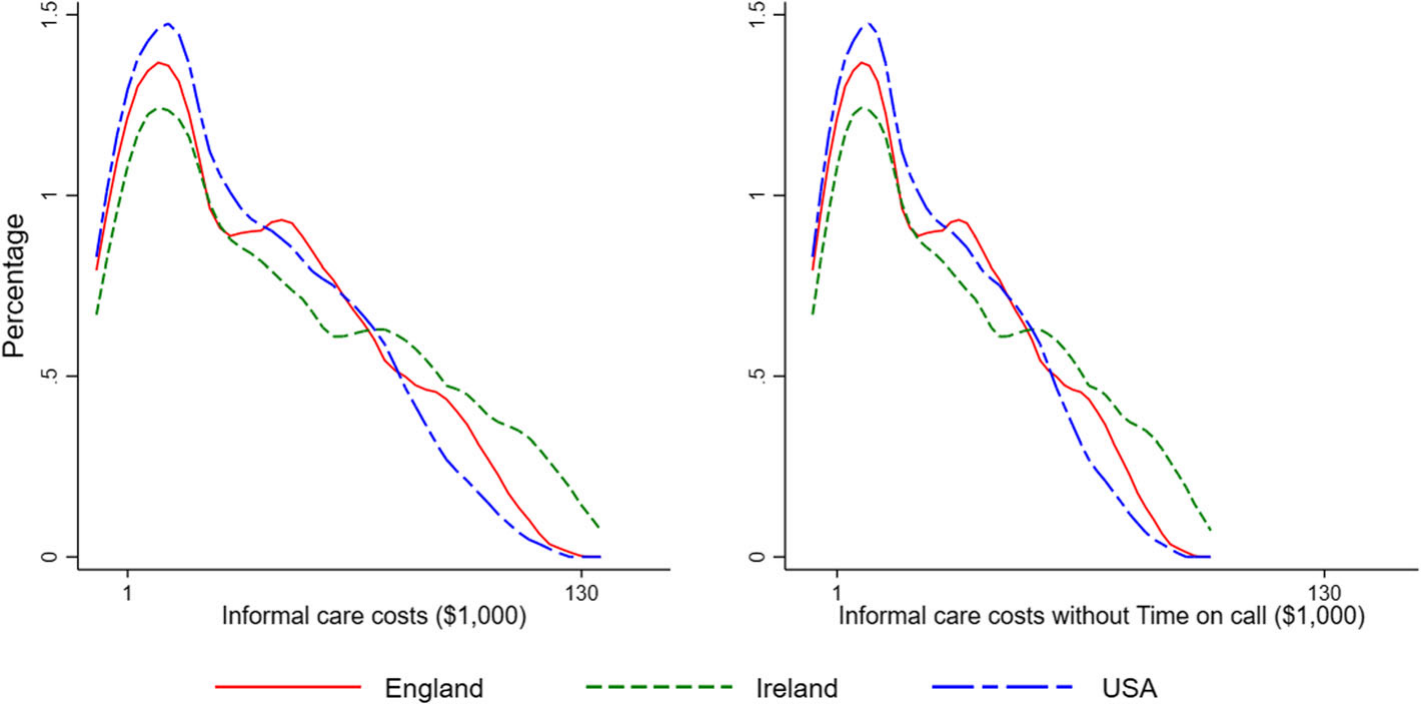
Distribution of costs of informal care (IC) provided for older patients in their last 3 months of life, with and without ‘time being on call’ in three countries

Median IC costs in the last 3 months of life were $28,847 (USA), $31,192 (England) and $36,930 (Ireland), with right skewed distributions that were similar for all countries (Fig. 1, Table 3, Additional file 1: Figure S2 and Figure S3). Removing the being on call element of IC cost estimates reduced the costs, although the distributions remained unchanged (Fig. 1, Table 3, Additional file 1: Figure S2 and Figure S3). IC costs varied less than did formal care costs between countries (Table 3). IC costs were not associated with total formal care costs (Pearson’s *r* = − 0.0057), nor with hospice/palliative care costs (Pearson’s *r* = 0.0608).

**Table 3:** Informal care (IC) costs and formal care costs of patients at the end of life in the last 3 months (US$)

	Informal care costs I			Informal care costs II*			Formal care costs^$^		Percentage of total care costs that are informal care costs	
	Mean	s.d.	Median	Mean	s.d.	Median	Mean	s.d.	Informal care costs I	Informal care costs II*
England	36,170	31,104	31,192	22,132	22,527	13,254	15,756	15,036	69.7%	58.4%
Ireland	43,760	36,930	36,398	26,767	25,477	18,604	29,210	24,231	60.0%	47.8%
USA	32,468	28,578	28,847	19,973	19,679	12,521	37,327	37,234	46.5%	34.9%
All countries	37,802	32,956	32,185	23,160	22,998	14,163	27,452	28,203	57.9%	45.8%

*Informal care cost II excludes the cost of “time spent ‘being on call’”

^$^Formal care costs were calculated by combining resource use data with unit costs obtained from standard as explained in [19]

IC costs were higher for husband/male partners ($48,679) and wife/female partners ($43,729) than those of daughters/sons or other relatives/others (Additional file 1: Table S1).

### Carer burden, positive aspects of caregiving (PAC), subsequent grief and informal care costs

Subsequent grief, carer burden and PAC were near normally distributed and were similar across the three countries (Additional file 1: Figure S1). Wives, husbands and daughters reported higher subsequent TRIG grief score than other relatives, implying lesser distress (Additional file 1: Table S2). Burden felt by carers was slightly higher among daughters and sons than others. Positive aspects of caregiving did not differ by the relationship to patient much except the male relatives.

**Fig. 2:**
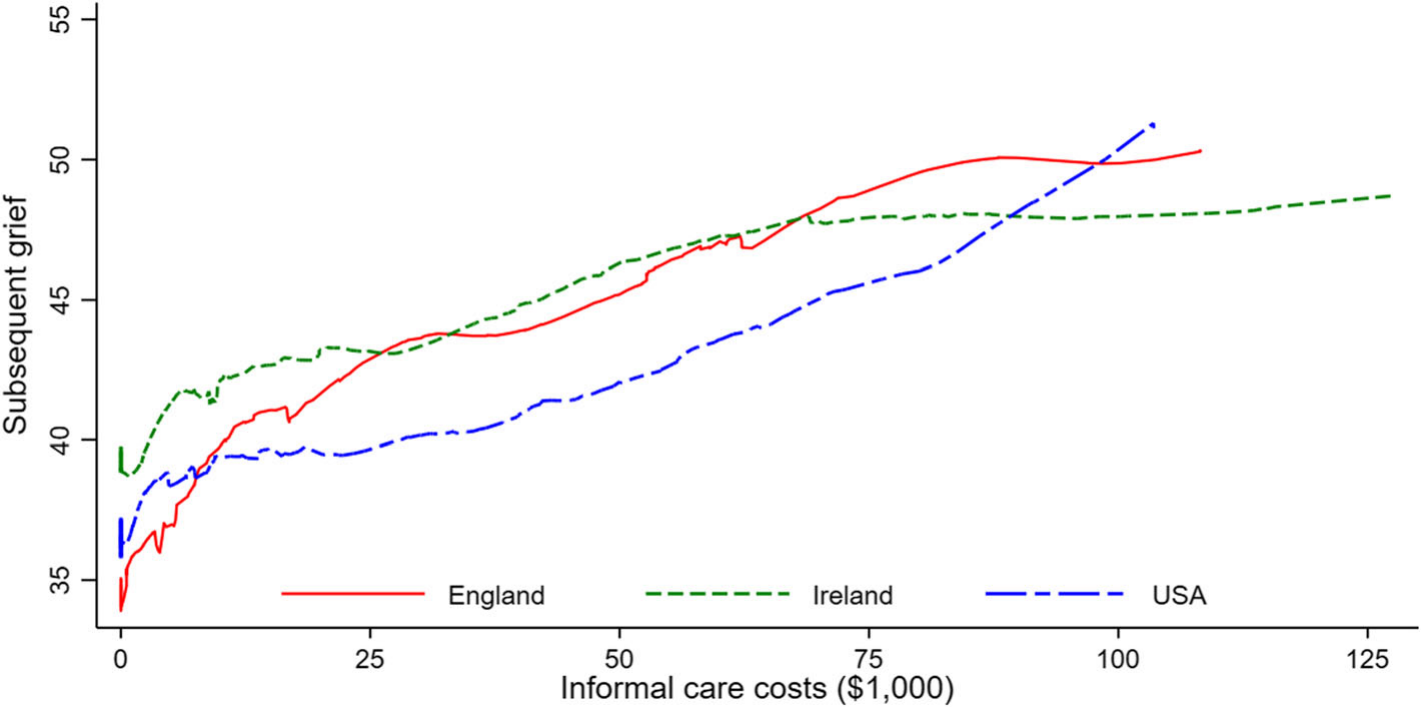
Relationship between subsequent grief and informal care (IC) costs in the last 3 months of older patient’s life in three countries. Note that higher grief scores indicate ‘less’ grief

In all three countries, there was a consistent pattern that subsequent grief was positively associated with IC costs, implying that more time spent on caring for patients was associated with lesser distress felt afterwards (Fig. 2).

### Multiple regression analysis

As for the univariate analysis, higher IC costs were associated with higher TRIG scores for subsequent grief, indicative of less grief (models 1 and 2, Table 4). However, the coefficients were small and the effects varied between individuals. For older patients, the carer’s subsequent grief score was lower, i.e. the carer grieved more.

**Table 4:** Factors associated with bereavement outcomes of carers who were identified as next of kin by older patients

	Subsequent grief		Burden		Positive aspects	
	(1)	(2)	(3)	(4)	(5)	(6)
Informal care cost ($1000)						
*β*	0.06*	0.06*	− 0.01	− 0.02	0.04*	0.03*
*CI*_*ll*_	0.03	0.03	− 0.03	− 0.04	0.02	0.01
*CI*_*up*_	0.09	0.10	0.01	0.01	0.06	0.06
Age at death						
*β*	− 0.23*	− 0.28*	− 0.04	− 0.01	0.02	0.00
*CI*_*ll*_	− 0.35	− 0.42	− 0.13	− 0.11	− 0.06	− 0.07
*CI*_*up*_	− 0.11	− 0.15	0.05	0.09	0.09	0.07
Cancer (= 1 if cancer)						
*β*	0.54	0.14	− 0.73	− 0.24	1.08	1.01
*CI*_*ll*_	− 1.46	− 2.15	− 2.25	− 2.02	− 0.17	− 0.43
*CI*_*up*_	2.54	2.43	0.78	1.55	2.32	2.45
Formal care cost ($1000)						
*β*	0.00	0.00	0.01	0.01	− 0.00	0.02
*CI*_*ll*_	− 0.03	− 0.05	− 0.01	− 0.02	− 0.02	− 0.00
*CI*_*up*_	0.04	0.05	0.03	0.05	0.02	0.05
Satisfaction with hospital care						
*β*	− 0.75^**^		− 0.76^**^		0.23	
*CI*_*ll*_	− 1.46		− 1.35		− 0.19	
*CI*_*up*_	− 0.05		− 0.17		0.65	
Satisfaction with home care						
*β*		− 0.47		− 0.84*		0.66*
*CI*_*ll*_		− 1.24		− 1.47		0.22
*CI*_*up*_		0.29		− 0.22		1.10
Carer religious (= 1 if religious)						
*β*	1.93	1.64	− 2.67^**^	− 4.01*	1.60^**^	1.32
*CI*_*ll*_	− 0.71	− 1.22	− 4.78	− 6.46	0.04	− 0.35
*CI*_*up*_	4.58	4.50	− 0.57	− 1.55	3.15	2.98
Relationship to patient (base: wife)						
Husband						
*β*	− 0.37	− 0.14	− 0.71	− 1.65	0.10	− 0.15
*CI*_*ll*_	− 3.54	− 3.88	− 2.94	− 4.25	− 1.99	− 2.58
*CI*_*up*_	2.80	3.61	1.53	0.96	2.19	2.27
Daughter						
*β*	0.92	1.51	3.85*	3.21*	0.90	0.55
*CI*_*ll*_	− 1.54	− 1.25	1.97	1.11	− 0.74	− 1.28
*CI*_*up*_	3.38	4.27	5.74	5.30	2.54	2.38
Son						
*β*	− 6.42^**^	− 3.92^**^	2.04	0.88	0.17	1.10
*CI*_*ll*_	− 9.51	− 7.34	− 0.61	− 2.07	− 1.88	− 1.13
*CI*_*up*_	− 3.32	− 0.51	4.69	3.83	2.21	3.34
Female relative						
*β*	− 6.20^**^	− 4.80	2.55	1.59	0.08	− 0.03
*CI*_*ll*_	− 10.65	− 10.11	− 0.44	− 2.11	− 2.24	− 2.82
*CI*_*up*_	− 1.74	0.51	5.53	5.30	2.40	2.77
Male relative						
*β*	− 14.02^**^	− 13.36*	− 1.68	− 3.66	− 2.62	0.53
*CI*_*ll*_	− 20.06	− 21.99	− 5.45	− 8.80	− 6.22	− 3.14
*CI*_*up*_	− 7.97	− 4.73	2.10	1.48	0.98	4.20
Others						
*β*	− 10.54^**^	− 11.08*	− 2.97	− 4.44^**^	1.24	2.01
*CI*_*ll*_	− 16.73	− 18.17	− 6.21	− 8.78	− 1.47	− 1.08
*CI*_*up*_	− 4.34	− 3.98	0.28	− 0.09	3.96	5.10
Carer’s financial status (base: living comfortably)						
Doing alright						
*β*	1.84	0.12	0.22	0.41	0.38	0.69
*CI*_*ll*_	− 0.29	− 2.26	− 1.31	− 1.37	− 0.93	− 0.76
*CI*_*up*_	3.97	2.50	1.75	2.19	1.70	2.15
Just about getting by						
*β*	3.94*	4.78*	1.38	3.15	− 0.50	− 0.64
*CI*_*ll*_	0.95	1.16	− 0.92	0.36	− 2.35	− 2.94
*CI*_*up*_	6.94	8.39	3.68	5.94	1.35	1.67
Finding it quite difficult						
*β*	7.91*	6.47*	3.06	3.72	− 2.05	− 0.74
*CI*_*ll*_	4.38	2.99	− 0.16	0.43	− 4.47	− 3.11
*CI*_*up*_	11.44	9.95	6.28	7.01	0.37	1.63
Finding it very difficult						
*β*	5.16	5.25	3.58	0.52	3.34	1.96
*CI*_*ll*_	− 6.31	− 10.48	− 1.24	− 4.73	− 1.71	− 2.84
*CI*_*up*_	16.63	20.98	8.40	5.78	8.39	6.75
Do not know						
*β*	3.20	2.58	− 1.45	− 2.03	0.83	1.22
*CI*_*ll*_	− 1.54	− 3.83	− 5.04	− 7.17	− 2.03	− 2.38
*CI*_*up*_	7.95	8.99	2.14	3.12	3.69	4.83
Country (base: England)						
Ireland						
*β*	0.22	0.47	− 1.54	− 1.41	0.86	− 0.36
*CI*_*ll*_	− 2.13	− 2.23	− 3.29	− 3.47	− 0.59	− 1.99
*CI*_*up*_	2.58	3.17	0.22	0.65	2.30	1.26
USA						
*β*	− 0.97	− 3.00^**^	0.09	− 0.20	1.92^**^	0.76
*CI*_*ll*_	− 3.51	− 5.86	− 1.82	− 2.31	0.38	− 0.88
*CI*_*up*_	1.57	− 0.15	1.99	1.92	3.47	2.40
Constant						
*β*	60.86*	64.11*	32.76*	32.32*	23.08*	22.67*
*CI*_*ll*_	50.04	51.53	24.54	23.11	16.42	15.68
*CI*_*up*_	71.67	76.70	40.99	41.53	29.74	29.66
Observations	608	459	609	459	604	456
*R*-squared	0.26	0.27	0.11	0.15	0.07	0.07

Notes: * *p* < 0.05, ** *p* < 0.1. Models 1, 3 and 5 included carer’s satisfaction with care in hospital, and models 2, 4 and 6 included carer’s satisfaction with care at home

Higher IC costs were also associated with higher scores for PAC (models 5 and 6, Table 2). Higher carer’s satisfaction with home care was associated with higher PAC.

IC costs were not associated with caregiver burden. Satisfaction with home care was negatively associated with caregiver burden, i.e. carers felt more burdened when they felt care provided for patients was not satisfactory (model 4, Table 4). Carers with a religious faith felt less burdened. Daughters felt more burdened, compared to wife/female partner of patients.

## Discussion

This is the first international study of ICrs of older people in their last 3 months of life. We found that IC costs were high ($37,802), representing 58% of total societal costs. Even when the elements of being on call are removed, IC costs still account for 46% of total care costs. Those carers who reported higher IC costs, and/or more hours of informal care, had lesser subsequent grief and reported more positive aspects of caregiving, without a negative effect on caregiver burden. Quality of care, as reported by the carer, was an important mediator; poorer experiences with home care were associated with more caregiver burden and fewer positive aspects of caregiving.

The inverse relationship between IC costs and subsequent grief surprised us. It appears that providing more hours of IC to patients protected the carer during bereavement, although the effect was small and varied. More hours of IC also led to a more positive feelings about caregiving. It may be that providing support protected ICrs from guilt in later bereavement. It may also be that more hours of IC support were provided by larger families and groups, and so the ICrs were not required to do so much individually, and possibly also gained from mutual support from other family members and friends. Other possible explanations include the following: that ICrs providing more support were more prepared for the death and ensured that the person they cared for did not feel burden to others; both factors possibly protect against complicated grief [34]. This finding needs more study, widening the usual approach of considering ‘single’ patient-family dyads. However, it is a promising development, as it suggests that ICrs can and often do want to be part of the caring team—and that this can be done without being harmful to them; they just need help to allow them to do this well. This finding should also inform the development of caregiver support interventions [35], which need to provide support across changes in setting [36]. Our finding did not support other meta-analyses that higher number of hours spent caregiving led to higher caregiver burden [32], and we suggest that this difference is because other studies did not account for variations in care quality and other potential confounders.

Poor formal home care quality was associated with poorer ICr outcomes, in terms of greater burden and fewer rewards. Poor quality of end-of-life care has been associated also with complicated grief in other population-based research [37]. Earlier analysis across these countries found that poor home care also was associated with high formal care costs [19]. In contrast, palliative care services had high quality, but were little used, accounting for only 1–15% of formal care costs [19]. Taken together, these findings suggest that improving community palliative care may improve care value, the care experience for patients and ICrs; increase IC rewards; and reduce IC burden and formal care costs. Treatments and interventions are ever more intricate, especially in the face of a multimorbid, older person, who is approaching the end of life [38, 39]. Co-ordination between settings and between the diversity of care interventions and treatments, communication and the response of staff in the face of clinical uncertainty collectively are vital to improve care experience, yet are often lacking [40, 41]. As in the closely related field of patient safely, consideration of the whole patient journey is vital [42]. Our finding is particularly important at a time when carers are being asked to do more, due to self- and family isolation for older people as a result of the COVID-19 pandemic [43], including potentially to administer medicines [44].

Interestingly, IC costs were quite similar between our countries, in contrast to formal end of life care costs which varied much more [19]. We found the contribution of ICrs in all three countries was similar in terms of hours of care provided and types of support given. Most studies of ICrs have considered the impacts on individual caregivers, rather than the needs of patients overall. A UK survey of bereaved cancer carers (29% response) found that respondents reported a median of 70 h of caregiving each week [45]. However, a societal perspective to supporting end of life care requires that the contribution of all caregivers be considered [46–48], as in our study. We observed that patients received higher levels of support, which is identified in earlier studies. Patients were provided with 19–21 h of IC per week for personal care, 15–18 h for medical procedures, 7–10 h for appointments and 17–21 h for household tasks. ICrs also spent 66–76 h per week on being on call and 52–55 h being with patients. Using the societal perspective taken, our data suggest that IC costs at the end of life (usually based on allocating a minimum wage to caregiver’s activities) account for more than half of total care costs. Out of pocket payment for medications and private health insurance are not included in this analysis, and so may slightly underestimate care costs. However, we do not believe that this substantially alters our findings. Payment for prescription medicines varies between countries so would limit international comparison. This descriptive data is an important contribution, because end of life care lags behind much of health care in economic appraisal [49]. We were able to identify some tasks that would likely need to be performed at a relatively fixed times of day (such as medical procedures, personal care, appointments), and others (such as household tasks) that may be adjustable and performed at other times or even on different days, reducing the ‘time-bound’ opportunity costs [50].

We were surprised by the amount of time spent by ICrs on call, this was the most common activity. It may be that the uncertainties encountered in end of life care [51–54] mean that ICrs felt that someone in the family was on call most of the time. This will require a flexibility by employers to allow ICrs to be able to respond to unpredictable needs. It also highlights the need for effective out-of-hours palliative care, in all settings, to support not only patients but caregivers, who are supporting patients throughout much of the week, and, at least in our study, anticipate problems outside ‘normal’ 9–5 working hours.

We were also surprised by the lower numbers of people with dementia in our study. However, this was a sample of people who were recruited from primarily hospital-based palliative care services, where people in late stage dementia may have limited access. Dementia may also have been under-represented in our data on primary diagnosis and is sometimes missed. This warrants further study, and we have planned the International, Access, Rights, and Empowerment II (IARE II), to study older people with symptoms and frailty who are not receiving specialist palliative care.

### Strengths

We took a societal approach to costing, including IC costs, which places greater recognition on the role of ICrs. We were able to collect the same data across our different countries, making the finding of similar patterns, in contrast to formal care costs which varied more between countries [19], more noteworthy and widely generalisable. We also had data from four major cities, all in the top ranks of the Global Power City Index: rankings are as follows: London 1, Dublin 33, New York 2 and San Francisco 18 [55], Cities are becoming the norm for many societies, and so our focus on cities makes our findings highly relevant to care for the future. We had a response similar to or better than similar mortality follow-back surveys [16, 56]. We focussed on the last 3 months of life, when it is known that formal care costs increase especially [7–10].

### Limitations

Our results are based on responses from bereaved carers or next of kin; thus, we do not know the informal care provided to patients who do not have such carers but might have had support from neighbours, friends or relatives missed by our survey. ICrs may have different perspectives from patients and may have recall bias about the care provided, although the time window used in our mortality follow-back survey, 4–10 months after bereavement, is usually considered optimal [16, 24]. Our respondents were identified by the specialist palliative care teams in participating hospitals which provided palliative care services for the bereaved carers. ICrs of patients who did not have access to palliative care or bereavement care may have different experiences, possibly worse. More than 80% of patients and ICrs reported having a religious belief, which is higher than might be expected. This may be due to sample or measurement bias. We do not know whether or how ICrs were practising beliefs. Thus, the results suggesting that ICrs with a religious belief felt less burdened should be treated with caution. We were not allowed under the ethics approval to collect data on non-responders, so we were not able to compare the characteristics of responders and non-responders. There were some differences between cities in their response rates, but the similarities between countries in informal care activities do not suggest that this altered our overall findings or conclusions. We asked about total IC activities involving all members of the family and friends, but the assessments of grief relate to the main ICrs, and for practical reasons, we were not able to study grief among all those involved in caregiving. The data are cross-sectional, which limits the basis for establishing causality: we cannot positively determine that increased IC costs protected against grief, nor that poor quality care resulted in greater IC burden or fewer rewards. However, our findings meet many of the Bradford-Hill criteria for supporting causal relationships, such as consistency with other literature and across settings, temporality, plausibility and coherence [57]. Further, in this complex situation of end of life care, it is impossible to understand any causal chain perfectly, i.e. know every factor that could be considered a cause [58, 59]. Even if the chain were clear, it would not be clear how best to change the outcome, as the interventions are by definition complex [18]. Our data provide insights into how to improve care value at the end of life, which is profoundly needed and can also help with the appropriate modelling of complex interventions [18]. Our data may also help with the development of robust business cases for palliative care [60].

## Conclusion

The contribution of ICrs is considerable, accounting for around 50% of total care costs. These costs are similar across countries. Training and support interventions for ICrs should target the wide range of activities that they undertake. Increased informal care hours and costs, can lead to more rewards and lesser subsequent grief. Therefore ICrs, including family and friends and beyond one main informal carer, are central at the end of life and should be considered in all interventions. Our finding of an association between poor care quality and poorer ICrs outcomes, including greater burden and fewer rewards, suggests an urgent need to improve care quality, through the better integration and support for dedicated community palliative care services, and support people across the whole journey of care. Improving community palliative care may improve care value, the care experience for patients and ICrs; increase IC rewards; and reduce IC burden and formal care costs and should be a focus for investment, including and importantly during the COVID-19 pandemic.

## Supplementary information

**Additional file 1: Table S1.** Informal care (IC) costs in the last three months of life categorized by the relationship of carers to patients. **Figure S1.** Distribution of subsequent grief, carer burden and positive aspects of caregiving and by country. **Table S2.** Subsequent grief, carer burden and positive feeling of care by carers’ relationship to patients. **Figure S2.** Histogram of costs of informal care (IC) provided for older patients in their last three months of life, with and without ‘Time being on call’ in three countries. **Figure S3.** Cumulative distribution of costs of informal care (IC) provided for older patients in their last three months of life, with and without ‘Time being on call’ in three countries.

## Data Availability

The anonymised datasets supporting the conclusions of this manuscript are available upon request to the corresponding authors and BuildCARE team.
