# Supplementary material for: Associations between informal care costs, care quality, carer rewards, burden and subsequent grief: the international, access, rights and empowerment mortality follow-back study of the last 3 months of life (IARE I study)
Source: BMC Med. 2020 Nov 3;18:344. doi: 10.1186/s12916-020-01768-7 (PMC7606031; doi:10.1186/s12916-020-01768-7)
Supplement: Supplementary file 1 — Additional file 1: Table S1. Informal care (IC) costs in the last three months of life categorized by the relationship of carers to patients. Figure S1. Distribution of subsequent grief, carer burden and positive aspects of caregiving and by country. Table S2. Subsequent grief, carer burden and positive feeling of care by carers’ relationship to patients. Figure S2. Histogram of costs of informal care (IC) provided for older patients in their last three months of life, with and without ‘Time being on call’ in three countries. Figure S3. Cumulative distribution of costs of informal care (IC) provided for older patients in their last three months of life, with and without ‘Time being on call’ in three countries. [file 12916_2020_1768_MOESM1_ESM.docx]

**Associations between informal care costs, care quality, carer rewards, burden and subsequent grief: the international, access, rights and empowerment mortality follow-back study of the last three months of life (IARE I study).**

*Higginson et al, Supplementary tables and figures*

Table S1. Informal care (IC) costs in the last three months of life categorized by the relationship of carers to patients.

| Relationship to patient | N | Informal care cost I | | Informal care cost II^*^ | |
| --- | --- | --- | --- | --- | --- |
|  |  | Mean | s.d. | Mean | s.d. |
| Wife | 172 | 43,726 | 33,756 | 27,797 | 27,797 |
| Husband | 94 | 48,697 | 31,546 | 28,953 | 28,953 |
| Daughter | 266 | 38,629 | 33,895 | 23,617 | 23,617 |
| Son | 90 | 28,415 | 28,767 | 17,326 | 17,326 |
| Female relative | 60 | 31,056 | 29,942 | 18,544 | 18,544 |
| Male relative | 24 | 17,475 | 20,850 | 9,728 | 9,728 |
| Others | 36 | 27,579 | 26,809 | 16,292 | 16,292 |

*Informal care cost II excluded the cost of “Time spent ‘being on call’.”

Figure S1. Distribution of subsequent grief, carer burden and positive aspects of caregiving and by country

Table S2. Subsequent grief, carer burden and positive feeling of care by carers’ relationship to patients.

| Relationship to patient | Subsequent grief | | | Carer burden | | | Positive feeling of care | | |
| --- | --- | --- | --- | --- | --- | --- | --- | --- | --- |
|  | N | mean | sd | N | mean | sd | N | mean | sd |
| Wife | 167 | 45.4 | 12.6 | 165 | 22.8 | 8 | 166 | 30.3 | 8.0 |
| Husband | 94 | 46.8 | 11.9 | 92 | 21.7 | 7.7 | 90 | 30.8 | 6.7 |
| Daughter | 262 | 45.9 | 10.9 | 262 | 26.9 | 9.2 | 261 | 30.2 | 6.3 |
| Son | 89 | 37.3 | 11.3 | 90 | 25.3 | 9.5 | 89 | 29.4 | 7.0 |
| Female relative | 58 | 37.6 | 14.4 | 58 | 24.2 | 9.3 | 56 | 29.4 | 7.8 |
| Male relative | 21 | 28.9 | 13.2 | 21 | 22.0 | 6.7 | 22 | 25.5 | 7.9 |
| Others | 35 | 30.7 | 14.8 | 34 | 21.1 | 7.6 | 33 | 30.0 | 6.1 |


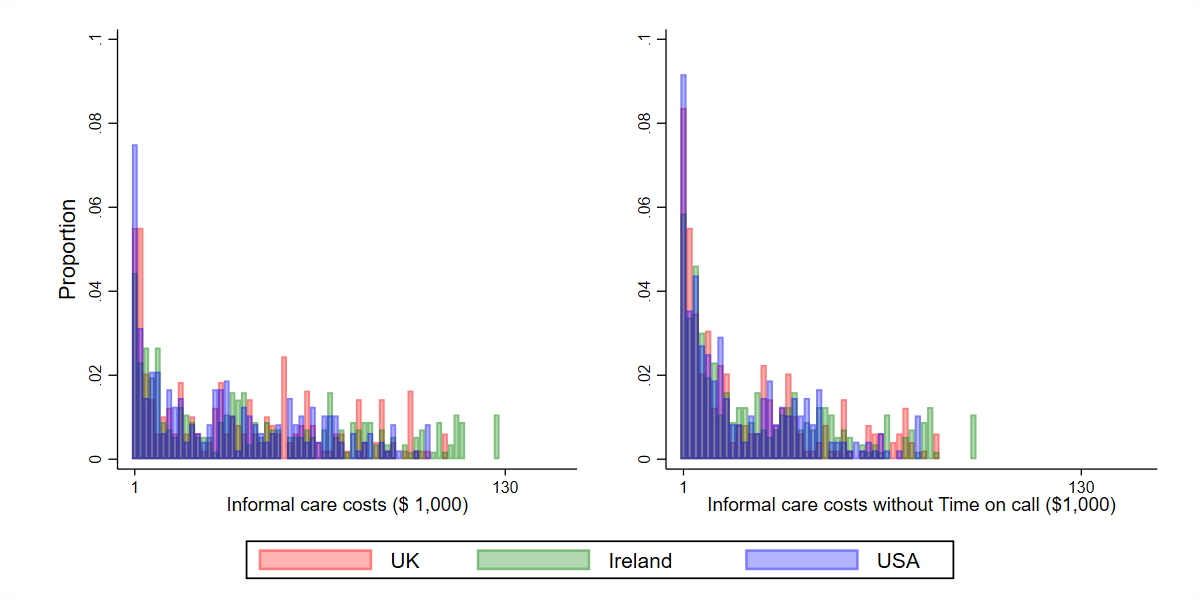


Figure S2. Histogram of costs of informal care (IC) provided for older patients in their last three months of life, with and without ‘Time being on call’ in three countries.


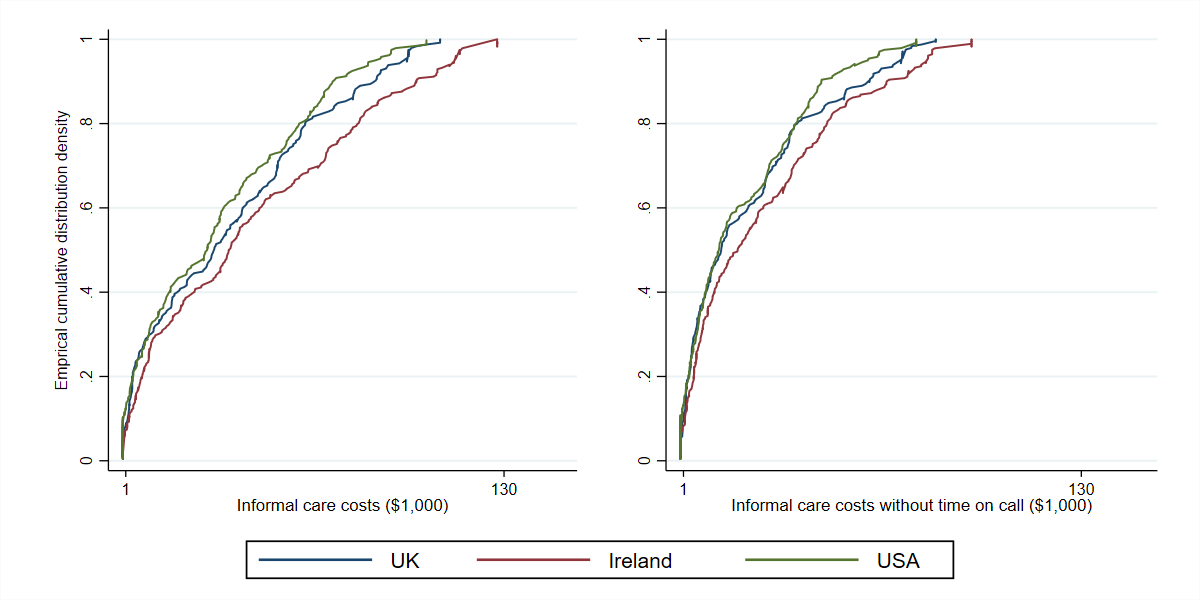


Figure S3. Cumulative distribution of costs of informal care (IC) provided for older patients in their last three months of life, with and without ‘Time being on call’ in three countries.
